# Equol status and changes in fecal microbiota in menopausal women receiving long-term treatment for menopause symptoms with a soy-isoflavone concentrate

**DOI:** 10.3389/fmicb.2015.00777

**Published:** 2015-08-05

**Authors:** Lucía Guadamuro, Susana Delgado, Begoña Redruello, Ana B. Flórez, Adolfo Suárez, Pablo Martínez-Camblor, Baltasar Mayo

**Affiliations:** ^1^Departamento de Microbiología y Bioquímica, Instituto de Productos Lácteos de Asturias – Consejo Superior de Investigaciones CientíficasVillaviciosa, Spain; ^2^Servicios Científico-Técnicos, Instituto de Productos Lácteos de Asturias – Consejo Superior de Investigaciones CientíficasVillaviciosa, Spain; ^3^Servicio de Digestivo, Hospital de CabueñesGijón, Spain; ^4^Consorcio de Apoyo a la Investigación Biomédica en Red, Hospital Universitario Central de AsturiasOviedo, Spain; ^5^Facultad de Ciencias de la Educación, Universidad Autónoma de ChileSantiago, Chile

**Keywords:** soy isoflavone, equol, intestinal microbiology, fecal microbiota, menopause, probiotics

## Abstract

The knowledge regarding the intestinal microbial types involved in isoflavone bioavailability and metabolism is still limited. The present work reports the influence of a treatment with isoflavones for 6 months on the fecal bacterial communities of 16 menopausal women, as determined by culturing and culture-independent microbial techniques. Changes in fecal communities were analyzed with respect to the women’s equol-producing phenotype. Compared to baseline, at 1 and 3 months the counts for all microbial populations in the feces of equol-producing women had increased strongly. In contrast, among the non-producers, the counts for all microbial populations at 1 month were similar to those at baseline, and decreased significantly by 3 and 6 months. Following isoflavone intake, major bands in the denaturing gradient gel electrophoresis (DGGE) profiles appeared and disappeared, suggesting important changes in majority populations. In some women, increases were seen in the intensity of specific DGGE bands corresponding to microorganisms known to be involved in the metabolism of dietary phytoestrogens (*Lactonifactor longoviformis*, *Faecalibacterium prausnitzii*, *Bifidobacterium* sp., *Ruminococcus* sp.). Real-Time quantitative PCR revealed that the *Clostridium leptum* and *C. coccoides* populations increased in equol producers, while those of bifidobacteria and enterobacteria decreased, and *vice versa* in the non-producers. Finally, the *Atopobium* population increased in both groups, but especially in the non-producers at three months. As the main findings of this study, (i) variations in the microbial communities over the 6-month period of isoflavone supplementation were large; (ii) no changes in the fecal microbial populations that were convincingly treatment-specific were seen; and (iii) the production of equol did not appear to be associated with the presence of, or increase in the population of, any of the majority bacterial types analyzed.

## Introduction

Compared to Caucasian women, fewer Asian women suffer discomfort during menopause. They also have better intestinal health, and enjoy lower rates of cardiovascular disease (Bhupathy et al., 2010) and cancer (Virk-Baker et al., 2010). The better health of these women has been associated with a higher intake of soy foods (Messina, 2000). Soy contains many biologically active compounds (Kang et al., 2010), but its beneficial health effects have been attributed to its isoflavone content (Sánchez-Calvo et al., 2013). At the molecular level, the health benefits of dietary isoflavones appear to be mediated through their hormonal (Yuan et al., 2007), antioxidant (Wang et al., 2008), and enzyme-inhibitory (Crozier et al., 2009) activities.

In nature, isoflavones (daidzin, genistin, glycitin) mostly (>80%) appear conjugated with sugars as isoflavone-glycosides, the bioavailability and bioactivity of which are low (Crozier et al., 2009; de Cremoux et al., 2010). For their full activity to be realized, aglycones (daidzein, genistein, glycitein) need to be released from these isoflavone-glycosides and, occasionally, metabolized (Sánchez-Calvo et al., 2013). The transformations necessary are mostly performed by the enzymes of the gut microbiota. However, though improving, our knowledge of the gut microbes, their enzymes, and the pathways involved in the metabolism of isoflavones, is still limited (Atkinson et al., 2005; Yuan et al., 2007; Kemperman et al., 2010; Clavel and Mapesa, 2013). The metabolism of isoflavones is thought to occur in sequential steps involving several enzymes produced by a number of microbial types (Clavel and Mapesa, 2013; Sánchez-Calvo et al., 2013). The release of aglycones from conjugated isoflavones starts via the action of the widely distributed glycosyl hydrolases (members of the β-glucosidase family; Cantarel et al., 2009). The aglycones formed then undergo dehydroxylation, reduction, the breakage of the pyrone ring, and demethylation, etc., giving rise to compounds either of greater biological activity (such as equol and 5-hydroxy equol) or inactive molecules [such as *o*-demethylangolensin (*O*-DMA); Clavel and Mapesa, 2013]. Due to the inter-individual diversity in microbiota composition (Frankenfeld, 2011), only around 30–50% of Western women are capable of producing equol, while around 80–90% produce *O*-DMA. It may be that equol-producing women benefit more fully from the intake of isoflavones (Sánchez-Calvo et al., 2013).

Like other polyphenols, isoflavones have antimicrobial activity, which can modulate the diversity and composition of the gut microbiota after their consumption (Kemperman et al., 2010). The inhibition of pathogens or an increase in the size of beneficial populations might then contribute toward health benefits. However, studies on how isoflavones influence the composition and activity of the gut microbial community, and its effect on human health, are scarce (Clavel et al., 2005; Bolca et al., 2007; Nakatsu et al., 2014). Further, the results of the studies that are available are hard to compare, a consequence of differences in treatment regimen, target group, and the techniques employed. However, understanding how microorganisms and metabolites interact and elicit a physiological response (or lack thereof) is crucial if the results of observational and interventional studies are to be properly interpreted. Investigations that assess *in vivo* the response of gut populations to isoflavone consumption are much needed.

The main aim of the present study was to determine the effect of long-term dietary supplementation with an isoflavone concentrate on the fecal microbial communities of menopausal women, via both culturing and culture-independent techniques. Women were entering a treatment of menopause symptoms with an isoflavone concentrate, which made unnecessary the design of an intervention study. The microbial results were correlated with equol production status in an attempt to identify those microbial populations and/or numbers linked to the production of this active, microbial-derived compound.

## Materials and Methods

### Human Volunteers and Urine and Stool Samples

This study was approved by the Research Ethics Committee of the Principado de Asturias, Spain. The selection of donors and later sampling was performed following standardized protocols recommended by the above committee. Sixteen menopausal women (age range 48–61; average 53.4) were recruited at the Obstetrics and Gynaecology Service of the Hospital de Cabueñes (Gijón, Spain). No participants suffered from any disease or intestinal disorder. Additionally, they had received no treatment with antibiotics or any other medication for at least 6 months prior to the start of the study. All participants consumed one tablet containing 80 mg of an isoflavone concentrate (Fisiogen; Zambon, Bresso, Italy) per day for 6 months. Urine and fecal samples were collected at four time points: before the start of the treatment (*t* = 0), and at one (*t* = 1), three (*t* = 3), and six (*t* = 6) months. Morning urine samples and freshly voided stools were collected by the volunteers themselves, the latter in sterile plastic containers, in which they were maintained under anaerobic conditions via the use of Anaerocult A (Merck, Darmstadt, Germany). All samples were a transported to the laboratory by courier. Stool samples were subjected to microbial analyses within 2 h of arrival; dilutions for culture-independent techniques were kept frozen at –80°C until use, as were urine samples for later equol and creatinine analysis.

### Determination of Equol and Creatinine in Urine Samples

Three milliliters of thawed urine samples were diluted with 3 mL of 0.1 M acetate buffer (pH 4.5) and treated for 20 h at 37°C with 10 μL of extract type H-1 crude enzyme solution from *Helix pomatia* (Sigma–Aldrich, St. Louis, MO, USA). This has β-glucuronidase (100 units/μL) and sulphatase (7.5 units/μL) activities. Equol was extracted using Bond Elut-C18 200 mg solid-phase cartridges (Agilent Technologies, Santa Clara, CA, USA), pre-conditioned with 3 mL of methanol and 3 mL of acetate buffer 0.1 M pH 4.5. Treated urine was passed through the cartridges, which were then washed with 3 mL acetate buffer 0.1 M (pH 4.5). To remove any residual water, 200 μL of ethyl acetate were eluted through the cartridges and rejected; this roughly corresponded to the column dead volume. Equol was then eluted with 1 mL of ethyl acetate, filtered through a 0.2 μm PTFE membrane, and then evaporated to dryness under vacuum at room temperature. Prior to analysis, extracts were dissolved in 100 μL methanol [high-performance liquid chromatography (HPLC) grade) and kept at 4°C in opaque vials with screw caps. A 2 mM stock solution of equol (Sigma–Aldrich) in methanol was used to prepare a calibration curve covering six concentrations from 0.005 to 363.60 μM.

Equol in urine was determined using an H-Class Acquity UPLC^TM^ ultra-high-performance liquid chromatography (UHPLC) system (Waters) equipped with a BEH reversed-phase C18 chromatographic column (1.7 μm, 2.1 mm × 100 mm; Waters, Palo Alto, CA, USA; Redruello et al., unpublished). The temperature of the column was set at 40°C. The mobile phase consisted of H_2_O supplemented with 0.05% phosphoric acid (solvent A) and 100% methanol (solvent B). Samples were applied onto the column and eluted at a flow rate of 0.45 mL/min according to the following linear gradient: 0 min 90% A/10% B; 1 min 88% A/12% B; 3.5 min 75% A/25% B; 6.5 min 75% A/25% B; 10 min 50% A/50% B; 12 min 10% A/90% B; and 16 min 10% A/90% B. This was followed by washing with 10%A/90% B for 4 min and then 5 min with 90% A/10% B to re-equilibrate the column. Equol was measured using a fluorescence detector (excitation 280 nm, emission 310 nm).

As a single urine sample was analyzed, creatinine was determined to normalize equol excretion values. Equol was measured using a kinetic-photometric method based on the Jaffe reaction (Heinegård and Tiderström, 1973). For this, urine was treated with an alkaline picrate solution resulting in a bright orange–red complex. The formation rate of the complex over a prefixed interval of time (measured as an increase in absorbance) is proportional to the concentration of creatinine in the sample. Reactions were performed in 96-well microplates and measurements made using a Benchmark Plus microplate spectrophotometer (Bio-Rad, Richmond, CA, USA). Two hundred microliters of 10-fold diluted urine were added to a 2 mL solution of 25 mM picric acid prepared in 300 mM phosphate buffer (pH 12.1) containing 2 g/L SDS (to avoid protein precipitation). The reaction was allowed to proceed at 37°C and mixed every 5 s. The absorbance at 510 nm was measured over 6 min and the reaction rate determined as the tangent in the linear part of the kinetic curve between 0.08 and 5 min. Each sample was assayed in triplicate.

### Fecal Microbiota Analyses

#### Microbial Counts

All fecal samples were processed in a Mac500 anaerobic chamber (Down Whitley Scientific, West Yorkshire, UK) containing a 10% H_2_, 10% CO_2_, 80% N_2_ atmosphere. For microbial analyses, 1 g of feces was homogenized in 9 mL of sterile Maximum Recovery Diluent (Scharlab, Barcelona, Spain). The homogenates were then serially diluted and plated in duplicate onto general and selective agar media. Total and indicator bacterial populations were counted using the media as follows: for lactobacilli, de Man-Rogosa-Sharpe agar (Scharlab) supplemented with 0.25% cysteine (VWR International, Radnor, PA, USA; MRSC); for clostridia, agarified Reinforced Clostridial Medium (RCM; Merck, Darmstadt, Germany); for *Bifidobacterium* sp., modified Columbia agar (BCCM^TM^/LMG, Medium M144; BIF; Masco et al., 2003); for Enterobacteriaceae, Eosin Methylene Blue agar (EMB; Oxoid, Basingstoke Hampshire, UK); for *Veillonella* sp., *Veillonella* agar (VA; Merck); for *Bacteroides* and *Prevotella* (BP), specialized *Bacteroides* and *Prevotella* agar (Ly et al., 2007); and for total microorganisms, Medium for Colon Bacteria (MCB; Van der Meulen et al., 2006). Counting was performed after anaerobic incubation of the plates at 37°C for 72 h, with the exception of the EMB plates, for which enumeration analysis was performed after aerobic incubation for 24 h. Data were recorded as colony forming units (cfu)/g of feces, were transformed to logarithmic units before statistical analysis.

#### DNA Extraction from fecal Samples

Extraction of total bacterial DNA was based on the method of Zoetendal et al. (2006) using the QIAamp DNA Stool Minikit (Qiagen, Hilden, Germany) with some modifications of the manufacturer’s protocol. Briefly, 0.2 g of thawed fecal samples were suspended in 1.8 mL of phosphate buffer saline solution (PBS; pH 7.4). The fecal suspension was homogenized by vortexing and centrifuged at 800 rpm at 4°C for 10 min to eliminate insoluble materials. Supernatants were transferred to new tubes and centrifuged at 14,000 rpm at 4°C for 5 min. Pellets were suspended in 200 μL of lysis solution (20 mM Tris-HCl pH 8.0, 2 mM EDTA, 1.20% Triton X-100, and 20 mg/mL lysozyme). Twenty units of mutanolysin (Merck, Darmstadt, Germany) were added to the mixture, which was then incubated at 37°C for 40 min. Immediately after, 1.2 mL of the lysis buffer from the DNA-isolation kit were added, the mixture placed in a screw-cap tube containing 0.3 and 0.1 g of 0.1 and 0.5 mm zirconia/silica beads, respectively, (BioSpec Products, Bartlesville, OK, USA) and subjected to mechanical breakage in a FastPrep FP120 Cell Disrupter (Qbiogene, Carlsbad, CA, USA; three cycles at 5.5 m s^-1^ for 30 s, cooling the samples on ice between cycles). Cell extracts were loaded onto the kit’s column following the manufacturer’s recommendations. Finally, the DNA was eluted with 150 μL sterile molecular biology grade water (Sigma–Aldrich) and stored at –20°C until required.

#### Denaturing Gradient Gel Electrophoresis (DGGE) Amplification

The variable V3 region of the 16S rRNA gene was amplified by PCR using the universal primers F357 (5′-TACGGGAGGCAGCAG-3′), to which a 39 bp GC sequence was linked to give rise to GC-F357 and R518 (5′-ATTACCGCGGCTGCTGG-3′), (Muyzer et al., 1993). The V2 and V4 regions were amplified with *Bifidobacterium*-specific primers F-Bif164 (5′-GGGTGGTAATGCCGGATG-3′) and R-Bif662 (5′-CCACCGTTACACCGGGAA-3′); a 40 bp GC sequence was linked to the latter to give rise to R-Bif662-GC (Satokari et al., 2001). Each reaction mixture consisted of 0.2 mM of each deoxynucleoside triphosphate (dNTPs), 0.24 μM of each forward and reverse primer, 2 U of 5 Prime Taq polymerase (VWR International), and between 100 and 150 ng of purified DNA. The PCR amplification programs were as follows: for primers F357-GC and R518 – an initial denaturation step at 95°C for 5 min, followed by 35 cycles of 95°C for 30 s, 56°C for 30 s, and 72°C for 40 s, plus a final extension step at 72°C for 10 min; for primers F-Bif164 and R-Bif662-G – after an identical denaturation step, 35 cycles of 95°C for 30 s, 62°C for 40 s, and 72°C for 1 min, plus a final extension step at 72°C for 5 min.

#### DGGE Analysis

Denaturing gradient gel electrophoresis was undertaken in a DCode apparatus (Bio-Rad) at 60°C. Electrophoresis was performed at 200 V for 10 min in an 8% polyacrylamide stacking gel, followed by 75 V for 16 h in a denaturing gel in 0.5X Tris-acetate-EDTA (TAE) buffer. The urea-formamide denaturing ranges were 40–55% for amplicons obtained with primers F357-GC/R518 and 45–55% for amplicons obtained with primers F-Bif164/R-Bif662-GC. After staining in an ethidium bromide solution, the gels were visualized under UV light using a GBox system (Syngene, Cambridge, UK) equipped with GeneSys image acquisition software (Syngene). Selected bands were excised from the gels, suspended in sterile molecular grade water, and kept overnight at 4°C. Subsequently, the eluted DNA was used as a template in new amplification reactions involving the same primers without the GC-clamp. Finally, amplicons were purified using GenElute PCR Clean-Up columns (Sigma–Aldrich) and sequenced using an ABI Prism gene sequencer (Applied Biosystems, Foster City, CA, USA). Sequences were compared using the Blast program^1^ and the Classifier tool provided by the Ribosomal Database Project^2^. Sequences with a percentage nucleotide match of 97% or higher to those in databases were assigned to the corresponding species.

The gels were digitalized and analyzed using Gene Tools v.4.03 software. For each DGGE profile, the Shannon–Weaver diversity index (H index) was estimated on the basis of the number of bands (assuming them to be equivalent to the number of species) and their relative intensity. Additionally, the similarity between the DGGE profiles (presence or absence of bands and their intensities) was determined by calculating the Dice’s coefficient. Clustering was performed using the unweighted pair group method with arithmetic averages (UPGMA), employing the DendroUPGMA computer program^3^.

#### Real-Time quantitative PCR (qPCR)

Quantification of the different bacterial populations in feces was performed by qPCR using group-specific primers targeting the 16S rRNA gene (**Table 1**). Amplification reactions were performed in 96-well optical plates (Applied Biosystems) in a 7500 Fast Real-Time PCR System (Applied Biosystems). All amplifications were performed in triplicate in a final volume of 25 μL containing 2x SYBR Green PCR Master Mix (Applied Biosystems), 0.2 μM of each primer and 1 μL of template DNA (5–10 ng). The thermal cycling protocol followed consisted of an initial cycle at 95°C for 10 min, followed by 40 cycles at 95°C for 15 s, and 1 min at the appropriate primer-pair annealing temperature (**Table 1**). To check for specificity, melting curve analysis was performed, increasing the temperature from 60 to 95°C at a rate of 0.2°C per second with the continuous monitoring of fluorescence. Primer efficiency was calculated from the slope of the standard curve for each primer set (*E* = 10^-1/slope^). The different bacterial groups were expressed as relative quantities (percentage of the total bacterial 16S rDNA in the sample) according to Vigsnæs et al. (2011).

**Table 1:** Bacterial target groups and characteristics of primers used for quantitative PCR (qPCR) in this study.

Microbial target	Primer	Sequence 5′–3′	Annealing (°C)	Efficiency^a^	Reference
*Bifidobacterium* sp.	F-bifido R-bifido	CGCGTCYGGTGTGAAAG CCCCACATCCAGCATCCA	60	1.90	Delroisse et al. (2008)
*Lactobacillus* sp.	Lacto-F Lacto-R	AGCAGTAGGGAATCTTCCA CACCGCTACACATGGAG	60	1.96	Heilig et al. (2002)
*Clostridium coccoides* group	g-Ccoc-F g-Ccoc-R	AAATGACGGTACCTGACTAA CTTTGAGTTTCATTCTTGCGAA	60	1.93	Matsuki et al. (2004)
*Clostridium leptum* group	g-Clept-F g-Clept-R	GCACAAGCAGTGGAGT CTTCCTCCGTTTTGTCAA	60	1.89	Matsuki et al. (2004)
*Bacteroidetes* phylum	Bact934F Bact1060R	GGARCATGTGGTTTAATTCGATGAT AGCTGACGACAACCATGCAG	60	1.92	Guo et al. (2008)
*Atopobium* cluster	c-Atopo-F c-Atopo-R	GGGTTGAGAGACCGACC CGGRGCTTCTTCTGCAGG	60	1.90	Matsuki et al. (2002)
Enterobacteriaceae	En-lsu3F En-lsu3-R	TGCCGTAACTTCGGGAGAAGGCA TCAAGGCTCAATGTTCAGTGTC	55	1.97	Matsuda et al. (2007)
Total bacteria^b^	TBA-F TBA-R	CGGCAACGAGCGCAACCC CCATTGTAGCACGTGTGTAGCC	60	1.93	Dennan and McSweeney (2006)

*^a^Primer efficiency was calculated from the slope of the standard curve for each primer set as *E* = 10^-1/slope^.*

*^b^TBA primers were used for quantification of total bacterial DNA. This value was used to normalize differences in DNA concentration between individual samples.*

### Statistical Analysis

Statistical analysis of the culturing and qPCR data was performed using free R software^4^. The Shapiro–Wilk test was used to check for the normal distribution of the data. As several variables did not follow a normal distribution, comparisons were performed by using non-parametric tests. The Mann–Whitney test for independent samples was performed to examine differences between equol producers and non-producers in terms of the microbial groups studied at every sampling point. The Wilcoxon signed-rank test for related samples was used to examine differences within microbial groups at all sampling points for both equol producers and non-producers. Two-tailed probability values of *P* < 0.05 were considered significant. Multivariate statistics was performed by principal coordinates analysis (PCoA) to search for associations between equol production status and the microbial groups determined by culturing and qPCR.

## Results

### Urine Equol after Isoflavone Supplementation

To test whether changes in the fecal microbial communities were associated with equol production phenotype, the equol status of the women was assessed by determining the concentration of this compound in their urine by UHPLC. Urine creatinine was also determined to normalize the equol values. Equol-producing women were defined as those responding to the soy challenge by showing an increase in urine equol to over 1000 nM (the cut-off defined by Rowland et al., 2000), and having an equol/creatinine ratio of >5.

Similar (and low) equol concentrations and equol/creatinine ratios were observed in the samples from all 16 women at *t* = 0 (**Table 2**). However, at *t* = 1, 100-fold increments in the ratios were obtained in the urine of four (25%) of the women (WA, WC, WG, and WP; Supplementary Table S1). In these subjects, the urinary excretion of equol at all post *t* = 0 sampling points (with the exception of *t* = 3 in woman WA) reached values above the stated cut-off. They were therefore considered equol producers. However, large variations in absolute equol concentrations were detected among their samples, as well as differences between samples for the same individual subject at different times (Supplementary Figure S1). In general, maximum equol production was observed at *t* = 1. After this point production was either maintained (in WC and WP) or fell by *t* = 3 and *t* = 6 (in WA and WG). Low equol levels, sometimes close to the limit of detection and/or of the limit of quantification, were measured in most urine samples from the non-producer women (**Table 2**).

**Table 2:** Average equol production during isoflavone treatment in urine samples among equol producer and non-producer women of this study.

Equol status	Sample (month)	Parameter	
		Equol^a^	Equol/Creatinine
Producers (*n* = 4)	0	59.50	0.58
	1	18716.75	77.62
	3	8114.00	38.13
	6	5702.50	28.63
Non-producers (*n* = 12)	0	39.00	0.73
	1	19.42	0.12
	3	28.08	0.23
	6	15.58	0.10

*^a^Concentration of equol in nM.*

### Microbial Counts

Wide inter-individual and inter-sample variations in counts for the different microbial populations were recorded over the supplementation period (**Table 3**). Although the response seemed erratic, some general trends were appreciated for equol producers and non-producers. At *t* = 0, counts for total and indicator microbial populations were generally slightly higher in the feces of the non-producers (**Table 3**). However, counts for all microbial populations increased strongly at *t* = 1 and *t* = 3 in the feces of the equol producers. In addition, in the equol producers, most cultivable populations decreased by *t* = 6, showing a trend toward the numbers recorded at *t* = 0. In contrast, in the non-producers, the counts for all microbial populations at *t* = 1 were similar to those at *t* = 0, but decreased significantly by *t* = 3 and *t* = 6 (**Table 3**). However, at the personal level, most microbial groups changed unpredictably in each of the women (Supplementary Table S2).

**Table 3:** Viable counts of total and indicator fecal microbial populations in menopausal women treated with a soy isoflavone supplement over a 6-month period.

Equol	Month	Microbial counts^a^						
		MCB	MRSC	BIF	RCM	EMB	BP	VA
Producers (*n* = 4)	0	9.51 ± 1.12	9.29 ± 1.39	9.44 ± 1.11	9.21 ± 1.31	7.69 ± 1.19	9.87 ± 1.27	9.65 ± 1.35
	1	10.46 ± 0.68	10.55 ± 0.82	10.28 ± 0.75	9.79 ± 1.92	8.91 ± 1.53	11.16 ± 0.63	10.65 ± 0.29
	3	10.73 ± 0.75	10.37 ± 0.83	10.49 ± 0.95	10.27 ± 0.52	7.35 ± 1.01	10.87 ± 0.87	10.42 ± 0.60
	6	9.79 ± 0.25	8.96 ± 0.87	9.53 ± 0.19	9.32 ± 0.40	7.17 ± 0.57	10.08 ± 0.13	9.36 ± 0.22
Non-producers (*n* = 12)	0	10.32 ± 0.61	10.04 ± 0.81	10.21 ± 0.67	10.23 ± 0.70	8.01 ± 0.98	10.45 ± 0.60	10.16 ± 0.73
	1	10.42 ± 0.81	10.06 ± 1.00	10.30 ± 0.82	10.38 ± 0.87	7.82 ± 0.70	10.80 ± 0.71	10.64 ± 0.75
	3	9.75 ± 0.76	9.30 ± 1.15	9.65 ± 0.69	9.49 ± 0.92	7.32 ± 1.07^∗^	10.13 ± 0.62	9.23 ± 0.85
	6	9.54 ± 0.54^∗∗^	9.10 ± 0.72^∗∗^	9.47 ± 0.49^∗∗^	9.19 ± 0.68^∗∗^	7.31 ± 1.03^∗^	9.74 ± 0.57^∗∗^	8.86 ± 0.75^∗∗∗^

*^a^Log_10_ cfu g^-1^ of feces (mean ± SD).*

*Key of media (target population): MCB, Medium for Colon Bacteria (total cultivable bacteria); MRSC; de Man, Rogosa, and Sharpe (lactobacilli); BIF, BCCMTM/LMG-Medium M144 (bifidobacteria); RCM, Reinforced Clostridium Medium (clostridia); EMB, Eosin Methylene Blue (enterobacterias); BP, Bacteroides and Prevotella medium (Bacteroides and Prevotella sp.); VA, Veillonella Agar (Veillonella sp.).*

*Key of statistical significance respect to basal time (t = 0): ^∗^p < 0.05; ^∗∗^p < 0.01; ^∗∗∗^p < 0.001.*

### Community Profiling by DGGE

**FIGURE 1:**
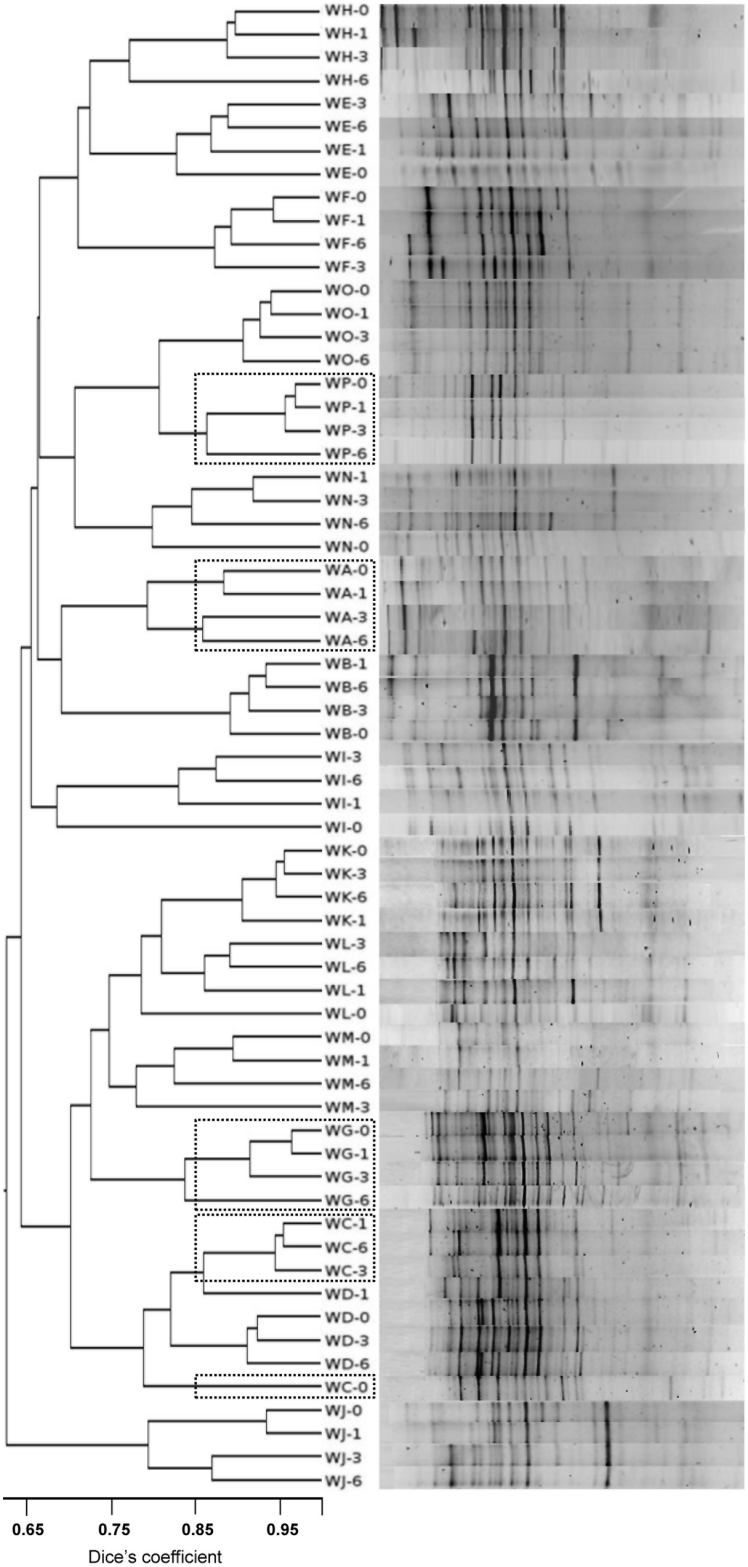
**Denaturing gradient gel electrophoresis (DGGE) profiles of the total microbial populations from fecal samples of menopausal women at different times during isoflavone treatment using universal prokaryotic primers amplifying the V2-V4 hypervariable regions of the 16S rRNA gene.** Similarity of the profiles was calculated by the Dice’s coefficient and clustered by the UPGMA method. Position of the profiles from equol producer women is indicated by a doted box.

The community profiles showed 12–22 distinct DGGE bands of different intensity (**Figure 1**). Profiles contained between 3 (WP samples) through 8 (WF, WH samples) bands of a high intensity, being the rest of low or very low intensity. The appearance and disappearance of intense bands in the samples of individual subjects at consecutive sampling points (see **Figure 1**, WD-1, WF-3, and WH-1) indicates major changes in the majority bacterial populations. Since DGGE is a semi-quantitative technique, increases and reductions in the intensity of bands suggests corresponding changes in the fortune of the associated species. In general, isoflavone supplementation led to a reduction in the Shannon–Weaver diversity index (H index) compared to *t* = 0. The UPGMA comparison of Dice’s coefficients showed a clear clustering of the profiles by subject (with only two exceptions, WC-0 and WD-1; **Figure 1**).

Eighty-two DGGE bands, the intensity of which varied over the supplementation period compared to *t* = 0, were assigned to bacterial types after DNA isolation, re-amplification, sequencing, and sequence comparison. The most common microbial types that responded to the presence of isoflavones were *Bifidobacterium adolescentis, Faecalibacterium prausnitzii, Lactonifactor longoviformis, Flavonifractor plautii, Coprococcus* sp., *Blautia* sp., *Oribacterium* sp., *Ruminococcus* sp., and members of the family Lachnospiraceae. However, the DGGE profiles from equol-producer women did not cluster together, and an apparent association between equol production and presence of specific bands was not observed.

Since the universal prokaryotic primers showed all bifidobacterial populations to increase over the supplementation period, DGGE analyses were performed using bifidobacteria-specific primers. Compared to the complex DGGE profiles for total bacteria, the bifidobacterial profiles were rather simple, involving just 2–5 bands per subject (partial results are provided in Supplementary Figure S2). In contrast to the general profiles, the bifidobacterial profiles for most of the women proved stable throughout the supplementation period. In total, 18 bands were isolated from the DGGE gels and identified as before. *B. bifidum, B. longum, B. catenulatum/B. pseudocatenulatum*, and *B. adolescentis* were the most common bifidobacterial species identified. Other species, such as *B. saeculare* and *B. ruminantium*, were occasionally identified. In two of the women (WB and WG), the bands corresponding to *B. adolescentis* increased markedly over the supplementation period.

### qPCR Analysis

qPCR detected wide variations in bacterial population sizes among subjects, and between samples from the same subject at different sampling points (Supplementary Table S3). The majority populations were formed by *Bacteroides* and *Clostridium*. Bifidobacteria and lactobacilli made up <10 and <0.5%, respectively, of total bacterial numbers. Over the supplementation period, opposite trends were seen between the equol producers and non-producers in terms of the change in some other bacterial populations. For example, bifidobacterial populations decreased in size in the equol producers, but increased in the non-producers, while the population of *Bacteroides* increase in equol producers and decrease in the non-producers (**Table 4**). The population of enterobacterias was shown to be significantly lower in equol producers than in non-producers. In this last group of women, increased numbers of enterobacteria were seen at *t* = 3 and *t* = 6 (**Table 4**). The two *Clostridium* clusters (leptum and coccoides) targeted by specific primers were seen to increase in the equol producers, especially at *t* = 3 and *t* = 6, while their numbers remained similar in samples from the non-producers. Finally, the *Atopobium* population increased over the supplementation period in both groups of women, but more so in the non-producers (a significant increase was detected at *t* = 3).

**Table 4:** Relative quantities of fecal microbial populations in the different equol status groups of the menopausal women treated with soy isoflavones of this study as determined by qPCR using universal and group-specific primers.

Equol status	Month	Microbial population^a^						
		Bifidobacteria	Lactobacilli	*Clostridium leptum*	*Clostridium coccoides*	*Bacteroides*	Enterobacteria	*Atopobium*
Producers (*n* = 4)	0	3.47 ± 5.74^a^	0.51 ± 0.91	28.70 ± 7.67	23.36 ± 14.31	73.6 ± 126.20	0.008 ± 0.001	4.87 ± 3.35


	1	1.00 ± 0.80	< 0.01 ± 0.01	19.64 ± 8.25	24.19 ± 18.58	104.9 ± 181.7	0.003 ± 0.004	5.64 ± 2.15
	3	2.05 ± 1.37	0.13 ± 0.20	35.69 ± 13.52	40.34 ± 24.18	45.31 ± 59.72	< 0.001 ± 0.001	5.96 ± 1.00
	6	1.43 ± 1.04	0.04 ± 0.03	33.14 ± 13.25	32.43 ± 24.08	113.3 ± 210.1	0.004 ± 0.006	4.70 ± 1.72
								
Non-producers (*n* = 12)	0	4.96 ± 6.19	0.31 ± 0.81	23.89 ± 8.75	25.33 ± 16.51	29.08 ± 63.03	0.20 ± 0.52	3.77 ± 1.97
	1	7.63 ± 7.87	0.31 ± 0.89	26.17 ± 17.11	26.97 ± 18.59	20.57 ± 31.07	0.14 ± 0.27	5.61 ± 4.25
	3	6.84 ± 9.17	0.05 ± 0.07	24.72 ± 10.89	25.15 ± 16.60	15.53 ± 14.42	0.83 ± 0.22	6.24 ± 4.90^∗^
	6	9.27 ± 13.11	0.35 ± 0.68	19.98 ± 10.13	24.26 ± 16.80	12.55 ± 9.46	0.49 ± 0.13	4.85 ± 2.88

*^a^Percentage of the total bacterial 16S rDNA (mean ± SD), as determined using the universal prokaryotic primers TBA-F and TBA-R (**Table 1**).*

*Key of statistical significance: ^∗^p < 0.05 versus basal sample (t = 0).*

## Discussion

The metabolism of soy isoflavones, and therefore their bioavailability and activity in humans, strongly depends on the activity of the intestinal microbiota. The underlying interactions between microbial populations and isoflavone metabolites, however, remain poorly understood (Frankenfeld, 2011; Clavel and Mapesa, 2013).

Though several methods have been proposed to determine human equol production phenotype (Rowland et al., 2000; Setchell and Cole, 2006), the cut-off for the assignment of producer or non-producer status remains rather arbitrary. In the present work, producers and non-producers were identified based on a cut-off of 1000 nM equol in urine, as defined by Rowland et al. (2000). The same number of equol producers and non-producers were also obtained when considering an equol/creatinine ratio >5 as the cut-off (Rowland et al., 2000). The frequency of equol producers differ widely among human communities (Setchell et al., 2002). That reported in the present work agrees well with values reported by other authors for Western women (Atkinson et al., 2005; Setchell and Cole, 2006; Possemiers et al., 2007; Frankenfeld, 2011; Nakatsu et al., 2014). In contrast to that reported by Franke et al. (2012), equol production phenotype proved to be stable over the study period; no conversion from equol producer to non-producer or *vice versa* was observed.

The effects of isoflavones on the gut microbiota have been little examined (Clavel et al., 2005; Bolca et al., 2007; Nakatsu et al., 2014), and with the exception of the study by Clavel et al. (2005), which lasted two months, changes in bacterial populations have only ever been monitored over short periods. Further, apart from the work of Nakatsu et al. (2014), which involved phylogenetic/metagenomic analyses, the number of populations targeted has been very limited. In the present work, the effects of isoflavones on the fecal microbiota of healthy menopausal women were analyzed at 1, 3, and 6 months of supplementation, and the results compared to those at baseline. This design, however, may have overlooked significant changes occurring soon after the start of isoflavone supplementation (Bolca et al., 2007; Nakatsu et al., 2014).

In general, the community structure and composition of the fecal bacterial populations changed significantly with the isoflavone supplementation. However, wide inter- and intra-individual (at different times) variations were detected by the different techniques employed, making it very difficult to correlate isoflavone intake with any changes in the bacterial community structure/population size, or changes in the latter with equol production. Thus, not surprisingly, PCoA of culturing and qPCR results showed no association between microbial communities and equol production status (Supplementary Figure S3). It is conceivable that isoflavones directly or indirectly affect members of the dominant microbiota (Clavel and Mapesa, 2013). In addition to the selective pressure they may exert on isoflavone-utilizing microorganisms, isoflavones or their metabolites (aglycones, equol, *O*-DMA, etc.) might modify conditions in the intestinal tract with an ensuing effect on susceptible bacterial communities. A bifidogenic effect of isoflavones has been reported in some studies (Clavel et al., 2005; Nakatsu et al., 2014), and a modest effect of this kind for most of the samples at *t* = 1 was confirmed by culturing. However, it did not persist through to *t* = 3 and *t* = 6. In equol producers, increases in population size within *Clostridium* clusters have been observed before (Clavel et al., 2005; Possemiers et al., 2007). However, such stimulatory effects may depend on the baseline sizes of these populations, which can vary widely between subjects. In contrast, a reduction was seen in the number of enterobacterias in the samples from most women (Supplementary Table S2). Since several members of the Enterobacteriaceae, including *Escherichia coli* phylotypes, harbor a ∼54 kb polyketide synthase (*pks*) pathogenicity island that encodes multi-enzymatic machinery for synthesizing a genotoxin that promotes tumorigenesis (Arthur et al., 2012), a fall in their numbers might be beneficial. However, no changes in the fecal microbial populations that were convincingly supplementation-specific were seen, suggesting that (at least some) changes might be due to normal variations caused by diet or other uncontrolled environmental factors. Together, the present enumeration results suggest that the consumption of isoflavones induces changes in the majority of microbial populations in both equol producers and non-producers, although these might be opposite.

The inadequacy of conventional culturing techniques for reflecting the microbial diversity of the intestinal ecosystem (Leser et al., 2002; Kemperman et al., 2010; Li et al., 2014) prompted the use of two culture-independent methods: DGGE for profiling the majority microbial species, and qPCR for targeting specific microbial populations. In some woman, specific bands corresponding to microorganisms previously associated with the metabolism of isoflavones and other dietary phytoestrogens (*L. longoviformis*, *F. prausnitzii*, *Bifidobacterium* sp., *Ruminococcus* sp.; Bolca et al., 2007; Clavel et al., 2007; Nakatsu et al., 2014) were enhanced in intensity. As suggested by Nakatsu et al. (2014), such increases may argue for isoflavones providing a chemical environment that selects for a subset of the initial bacterial community (Clavel and Mapesa, 2013). Alternatively, isoflavones might have antimicrobial effects on certain bacterial populations, as reported for flavonoid compounds (Orhan et al., 2010). In either case, the UPGMA analysis grouped the DGGE profiles by individual rather than by treatment (isoflavones, non-isoflavones), suggesting the changes detected by this technique depend on the initial microbial profile (personal microbiota). Furthermore, DGGE only detects changes occurring within the dominant bacterial populations (Muyzer et al., 1993); subtle changes in subdominant or minority species also occur but go unseen, and equol production might result from interactions between dominant and subdominant (or even minority) intestinal populations. In contrast, qPCR involving universal and group-specific primers produces a general microbial picture similar to that produced by state-of-the-art metagenomic techniques (Qin et al., 2010; Li et al., 2014).

Discrepancies between culturing and culture-independent methods may result from differences in lysis efficiency during DNA isolation, preferential PCR amplification (which may be different for each primer pair), or interspecies differences in 16S rRNA operon copy number (Kemperman et al., 2010). The detection of non-cultivatable and dead cells by DNA-based techniques may account for further differences. In this sense, low recoveries of Bacteroidetes from frozen fecal samples have been reported (Bahl et al., 2012). However, in the present work *Bacteroides* sp. proved to be majority population in both equol producers and non-producers.

## Conclusion

Both the culturing and the culture-independent methods used in this work detected wide microbial diversity in the studied menopausal women at baseline. Variations in the microbial communities over the six-month period of isoflavone supplementation were also large. No general patters of change due to isoflavone ingestion, or associated with equol production, were observed. The production of equol could not be correlated to the presence of, or increase in, any of the bacterial populations analyzed. This suggests isoflavone metabolism may differ in different people depending on their personal gut microbiota. Metagenomics, metabolomics, and metatranscriptomics analyses will be required to uncover the relationships between the structure and composition of the intestinal microbial communities in response to soy isoflavones and their metabolic compounds. Further research in this area could ultimately lead to the modulation of numbers of equol-producing bacteria, and thus equol producer-status, through the use of specific prebiotics and/or probiotics.

## Conflict of Interest Statement

The authors declare that the research was conducted in the absence of any commercial or financial relationships that could be construed as a potential conflict of interest.

## Footnotes

1 http://www.ncbi.nlm.nih.gov/BLAST/

2 http://rdp.cme.msu.edu/index.jsp

3 http://genomes.urv.es/UPGMA/

4 http://www.r-project.org
