# Supplementary material for: Equol status and changes in fecal microbiota in menopausal women receiving long-term treatment for menopause symptoms with a soy-isoflavone concentrate
Source: Front Microbiol. 2015 Aug 5;6:777. doi: 10.3389/fmicb.2015.00777 (PMC4525046; doi:10.3389/fmicb.2015.00777)
Supplement: Supplementary file 1 [file Data_Sheet_1.DOCX]

**Supplementary Table 1.-** Equol concentration during isoflavone treatment in urine samples of all 16 women in this study.

| Woman | Sample | Parameter | | |
| --- | --- | --- | --- | --- |
|  |  | Equol^a^ | Creatinine^b^ | Equol/Creatinine |
|  |  |  |  |  |
| **WA** | 0 | 165 | 106 | 1.56 |
|  | 1 | 32006 | 256 | 124.78 |
|  | 3 | 389 | 166 | 2.34 |
|  | 6 | 1379 | 179 | 7.70 |
|  |  |  |  |  |
| **WB** | 0 | 47 | 204 | 0.23 |
|  | 1 | 81 | 302 | 0.27 |
|  | 3 | 80 | 199 | 0.40 |
|  | 6 | 41 | 182 | 0.22 |
|  |  |  |  |  |
| **WC** | 0 | 9 | 94 | 0.10 |
|  | 1 | 1143 | 64 | 17.87 |
|  | 3 | 1727 | 168 | 10.29 |
|  | 6 | 1382 | 220 | 6.28 |
|  |  |  |  |  |
| **WD** | 0 | 2 | 155 | 0.01 |
|  | 1 | 2 | 60 | 0.03 |
|  | 3 | 9 | 158 | 0.06 |
|  | 6 | nd | 99 | nd |
|  |  |  |  |  |
| **WE** | 0 | nd | 163 | nd |
|  | 1 | nd | 259 | nd |
|  | 3 | nd | 126 | nd |
|  | 6 | nd | 146 | nd |
|  |  |  |  |  |
| **WF** | 0 | <1.1 | 85 | <1.1 |
|  | 1 | nd | 120 | nd |
|  | 3 | <1.1 | 263 | <1.1 |
|  | 6 | <1.1 | 230 | <1.1 |
|  |  |  |  |  |
| **WG** | 0 | 58 | 92 | 0.64 |
|  | 1 | 39965 | 246 | 162.70 |
|  | 3 | 28829 | 215 | 133.85 |
|  | 6 | 18741 | 250 | 75.11 |
|  |  |  |  |  |
| **WH** | 0 | 22 | 73 | 0.30 |
|  | 1 | 24 | 89 | 0.27 |
|  | 3 | 19 | 72 | 0.27 |
|  | 6 | 22 | 108 | 0.20 |
|  |  |  |  |  |
| **WI** | 0 | 20 | 88 | 0.23 |
|  | 1 | 50 | 316 | 0.16 |
|  | 3 | - | - | - |
|  | 6 | 37 | 149 | 0.25 |
|  |  |  |  |  |
| **WJ** | 0 | nd | 130 | nd |
|  | 1 | 13 | 169 | 0.08 |
|  | 3 | 171 | 115 | 1.49 |
|  | 6 | 54 | 160 | 0.34 |
|  |  |  |  |  |
| **WK** | 0 | <1.1 | 37 | <1.1 |
|  | 1 | 3 | 58 | 0.05 |
|  | 3 | 4 | 120 | 0.03 |
|  | 6 | <1.1 | 75 | <1.1 |
|  |  |  |  |  |
| **WL** | 0 | <1.1 | 94 | <1.1 |
|  | 1 | <1.1 | 117 | <1.1 |
|  | 3 | <1.1 | 156 | <1.1 |
|  | 6 | 4 | 206 | 0.02 |
|  |  |  |  |  |
| **WM** | 0 | <1.1 | 128 | <1.1 |
|  | 1 | 55 | 98 | 0.56 |
|  | 3 | 9 | 73 | 0.13 |
|  | 6 | 4 | 114 | 0.03 |
|  |  |  |  |  |
| **WN** | 0 | 377 | 47 | 7.97 |
|  | 1 | 0 | 127 | 0.00 |
|  | 3 | 23 | 81 | 0.29 |
|  | 6 | 18 | 292 | 0.06 |
|  |  |  |  |  |
| **WO** | 0 | <1.1 | 202 | <1.1 |
|  | 1 | 5 | 206 | 0.03 |
|  | 3 | 22 | 314 | 0.07 |
|  | 6 | 7 | 152 | 0.04 |
|  |  |  |  |  |
| **WP** | 0 | 6 | 257 | 0.03 |
|  | 1 | 1753 | 341 | 5.14 |
|  | 3 | 1511 | 250 | 6.05 |
|  | 6 | 1308 | 51 | 25.44 |
|  |  |  |  |  |

Concentration: ^a^nM of equol; ^b^M of creatinine.

nd, not detected.

**Supplementary Table 2.-** Viable counts of total and indicator faecal microbial populations in postmenopausal women treated with soy isoflavones over a six-month medication.

| Woman | Month | Microbial counts^a^ | | | | | | |
| --- | --- | --- | --- | --- | --- | --- | --- | --- |
|  |  | MCB | MRSC | BIF | RCM | EMB | BP | VA |
|  |  |  |  |  |  |  |  |  |
| **WA** | 0 | 8.85E+00 | 8.16E+00 | 8.83E+00 | 8.08E+00 | 6.48E+00 | 9.09E+00 | 8.77E+00 |
|  | 1 | 9.70E+00 | 9.48E+00 | 9.18E+00 | 9.97E+00 | 9.08E+00 | 1.07E+01 | 1.03E+01 |
|  | 3 | 1.16E+01 | 1.10E+01 | 1.17E+01 | 1.08E+01 | 8.59E+00 | 1.18E+01 | 1.07E+01 |
|  | 6 | 9.68E+00 | 7.70E+00 | 9.60E+00 | 8.78E+00 | 6.99E+00 | 9.95E+00 | 9.48E+00 |
|  |  |  |  |  |  |  |  |  |
| **WB** | 0 | 1.09E+01 | 1.10E+01 | 1.10E+01 | 1.08E+01 | 9.24E+00 | 1.09E+01 | 1.10E+01 |
|  | 1 | 1.16E+01 | 1.14E+01 | 1.16E+01 | 1.12E+01 | 7.77E+00 | 1.13E+01 | 1.15E+01 |
|  | 3 | 1.13E+01 | 1.13E+01 | 1.11E+01 | 1.11E+01 | 8.12E+00 | 1.12E+01 | 1.11E+01 |
|  | 6 | 1.02E+01 | 9.99E+00 | 1.02E+01 | 1.01E+01 | 9.01E+00 | 1.03E+01 | 1.01E+01 |
|  |  |  |  |  |  |  |  |  |
| **WC** | 0 | 1.06E+01 | 1.05E+01 | 1.05E+01 | 1.05E+01 | 9.29E+00 | 1.04E+01 | 9.70E+00 |
|  | 1 | 1.05E+01 | 1.15E+01 | 1.06E+01 | 7.02E+00 | 1.08E+01 | 1.07E+01 | 1.06E+01 |
|  | 3 | 1.11E+01 | 1.10E+01 | 1.08E+01 | 1.06E+01 | 6.64E+00 | 1.14E+01 | 1.10E+01 |
|  | 6 | 1.01E+01 | 9.61E+00 | 9.75E+00 | 9.67E+00 | 6.98E+00 | 1.00E+01 | 9.18E+00 |
|  |  |  |  |  |  |  |  |  |
| **WD** | 0 | 1.04E+01 | 1.06E+01 | 1.05E+01 | 1.04E+01 | 7.15E+00 | 1.05E+01 | 1.06E+01 |
|  | 1 | 1.16E+01 | 1.12E+01 | 1.14E+01 | 1.11E+01 | 8.58E+00 | 1.14E+01 | 1.12E+01 |
|  | 3 | 9.24E+00 | 9.73E+00 | 9.45E+00 | 9.63E+00 | 6.67E+00 | 9.97E+00 | 9.00E+00 |
|  | 6 | 9.24E+00 | 9.18E+00 | 9.32E+00 | 9.39E+00 | 7.39E+00 | 9.32E+00 | 8.00E+00 |
|  |  |  |  |  |  |  |  |  |
| **WE** | 0 | 9.31E+00 | 9.33E+00 | 9.47E+00 | 9.27E+00 | 8.86E+00 | 9.94E+00 | 8.88E+00 |
|  | 1 | 1.03E+01 | 9.66E+00 | 9.75E+00 | 9.72E+00 | 7.42E+00 | 1.03E+01 | 1.02E+01 |
|  | 3 | 9.75E+00 | 9.24E+00 | 9.20E+00 | 9.56E+00 | 8.41E+00 | 9.74E+00 | 8.00E+00 |
|  | 6 | 9.58E+00 | 9.49E+00 | 9.63E+00 | 9.73E+00 | 6.93E+00 | 1.05E+01 | 8.65E+00 |
|  |  |  |  |  |  |  |  |  |
| **WF** | 0 | 1.05E+01 | 9.02E+00 | 9.33E+00 | 9.44E+00 | 6.48E+00 | 1.03E+01 | 9.91E+00 |
|  | 1 | 9.33E+00 | 9.87E+00 | 1.00E+01 | 1.01E+01 | 7.16E+00 | 1.03E+01 | 1.02E+01 |
|  | 3 | 9.54E+00 | 7.70E+00 | 9.31E+00 | 8.00E+00 | 6.57E+00 | 1.02E+01 | 8.40E+00 |
|  | 6 | 9.82E+00 | 9.04E+00 | 9.25E+00 | 8.00E+00 | 8.15E+00 | 1.01E+01 | 9.41E+00 |
|  |  |  |  |  |  |  |  |  |
| **WG** | 0 | 8.28E+00 | 8.00E+00 | 8.20E+00 | 8.08E+00 | 7.74E+00 | 8.58E+00 | 8.60E+00 |
|  | 1 | 1.13E+01 | 1.06E+01 | 1.08E+01 | 1.11E+01 | 7.08E+00 | 1.13E+01 | 1.09E+01 |
|  | 3 | 1.03E+01 | 9.33E+00 | 9.79E+00 | 9.84E+00 | 6.42E+00 | 1.04E+01 | 1.02E+01 |
|  | 6 | 9.76E+00 | 9.47E+00 | 9.49E+00 | 9.57E+00 | 6.69E+00 | 1.03E+01 | 9.61E+00 |
|  |  |  |  |  |  |  |  |  |
| **WH** | 0 | 1.04E+01 | 1.02E+01 | 1.06E+01 | 1.07E+01 | 9.26E+00 | 1.07E+01 | 9.70E+00 |
|  | 1 | 1.05E+01 | 1.05E+01 | 1.07E+01 | 1.06E+01 | 8.60E+00 | 1.18E+01 | 1.15E+01 |
|  | 3 | 1.08E+01 | 1.07E+01 | 1.07E+01 | 1.08E+01 | 8.42E+00 | 1.15E+01 | 9.45E+00 |
|  | 6 | 1.03E+01 | 9.44E+00 | 1.01E+01 | 9.48E+00 | 8.17E+00 | 1.04E+01 | 9.15E+00 |
|  |  |  |  |  |  |  |  |  |
| **WJ** | 0 | 1.00E+01 | 1.01E+01 | 9.95E+00 | 1.02E+01 | 6.84E+00 | 1.06E+01 | 1.02E+01 |
|  | 1 | 1.06E+01 | 1.04E+01 | 1.04E+01 | 1.07E+01 | 7.24E+00 | 1.09E+01 | 1.05E+01 |
|  | 3 | 9.95E+00 | 9.60E+00 | 9.57E+00 | 9.34E+00 | 6.84E+00 | 9.82E+00 | 8.70E+00 |
|  | 6 | 9.87E+00 | 9.74E+00 | 9.66E+00 | 9.61E+00 | 7.46E+00 | 9.85E+00 | 9.30E+00 |
|  |  |  |  |  |  |  |  |  |
| **WI** | 0 | 9.98E+00 | 9.80E+00 | 9.70E+00 | 9.78E+00 | 7.64E+00 | 9.87E+00 | 9.82E+00 |
|  | 1 | 1.03E+01 | 9.65E+00 | 9.72E+00 | 1.02E+01 | 7.96E+00 | 1.04E+01 | 1.04E+01 |
|  | 3 | 1.02E+01 | 9.84E+00 | 9.96E+00 | 9.83E+00 | 6.68E+00 | 1.03E+01 | 1.04E+01 |
|  | 6 | 9.64E+00 | 9.57E+00 | 9.57E+00 | 9.57E+00 | 5.00E+00 | 9.51E+00 | 9.33E+00 |
|  |  |  |  |  |  |  |  |  |
| **WK** | 0 | 1.06E+01 | 1.07E+01 | 1.05E+01 | 1.07E+01 | 7.86E+00 | 1.12E+01 | 1.08E+01 |
|  | 1 | 1.03E+01 | 9.74E+00 | 9.54E+00 | 1.02E+01 | 8.23E+00 | 1.01E+01 | 1.03E+01 |
|  | 3 | 9.40E+00 | 8.93E+00 | 9.26E+00 | 9.12E+00 | 8.16E+00 | 9.69E+00 | 9.59E+00 |
|  | 6 | 9.71E+00 | 9.49E+00 | 9.75E+00 | 9.75E+00 | 6.70E+00 | 9.88E+00 | 9.81E+00 |
|  |  |  |  |  |  |  |  |  |
| **WL** | 0 | 1.15E+01 | 1.14E+01 | 1.15E+01 | 1.16E+01 | 9.00E+00 | 1.15E+01 | 1.15E+01 |
|  | 1 | 9.39E+00 | 9.19E+00 | 9.53E+00 | 1.10E+01 | 7.00E+00 | 1.13E+01 | 1.13E+01 |
|  | 3 | 9.27E+00 | 9.88E+00 | 1.01E+01 | 9.90E+00 | 8.26E+00 | 1.04E+01 | 9.15E+00 |
|  | 6 | 9.65E+00 | 9.22E+00 | 9.76E+00 | 9.32E+00 | 8.04E+00 | 9.96E+00 | 8.00E+00 |
|  |  |  |  |  |  |  |  |  |
| **WM** | 0 | 1.02E+01 | 9.92E+00 | 1.03E+01 | 1.04E+01 | 8.02E+00 | 9.73E+00 | 9.96E+00 |
|  | 1 | 1.10E+01 | 1.07E+01 | 1.10E+01 | 1.09E+01 | 6.71E+00 | 1.07E+01 | 1.03E+01 |
|  | 3 | 9.47E+00 | 9.28E+00 | 9.33E+00 | 9.71E+00 | 5.48E+00 | 9.71E+00 | 9.33E+00 |
|  | 6 | 8.40E+00 | 7.70E+00 | 8.72E+00 | 8.70E+00 | 6.60E+00 | 9.15E+00 | 7.88E+00 |
|  |  |  |  |  |  |  |  |  |
| **WN** | 0 | 9.33E+00 | 8.60E+00 | 9.46E+00 | 9.22E+00 | 8.63E+00 | 9.56E+00 | 9.34E+00 |
|  | 1 | 9.18E+00 | 7.70E+00 | 9.07E+00 | 0.00E+00 | 8.41E+00 | 9.45E+00 | 8.95E+00 |
|  | 3 | 8.40E+00 | 7.70E+00 | 8.74E+00 | 8.18E+00 | 8.31E+00 | 9.46E+00 | 8.74E+00 |
|  | 6 | 8.94E+00 | 8.30E+00 | 8.74E+00 | 8.30E+00 | 7.59E+00 | 8.68E+00 | 8.40E+00 |
|  |  |  |  |  |  |  |  |  |
| **WO** | 0 | 1.07E+01 | 9.82E+00 | 1.02E+01 | 1.03E+01 | 7.15E+00 | 1.06E+01 | 1.02E+01 |
|  | 1 | 1.10E+01 | 1.07E+01 | 1.09E+01 | 1.09E+01 | 8.75E+00 | 1.16E+01 | 1.14E+01 |
|  | 3 | 9.64E+00 | 7.70E+00 | 9.09E+00 | 8.76E+00 | 5.93E+00 | 9.61E+00 | 8.93E+00 |
|  | 6 | 9.08E+00 | 8.00E+00 | 8.92E+00 | 8.30E+00 | 6.64E+00 | 9.21E+00 | 8.30E+00 |
|  |  |  |  |  |  |  |  |  |
| **WP** | 0 | 1.03E+01 | 1.05E+01 | 1.02E+01 | 1.01E+01 | 7.26E+00 | 1.14E+01 | 1.15E+01 |
|  | 1 | 1.03E+01 | 1.07E+01 | 1.05E+01 | 1.11E+01 | 8.68E+00 | 1.20E+01 | 1.09E+01 |
|  | 3 | 9.94E+00 | 1.01E+01 | 9.67E+00 | 9.79E+00 | 7.75E+00 | 9.91E+00 | 9.67E+00 |
|  | 6 | 9.57E+00 | 9.08E+00 | 9.29E+00 | 9.27E+00 | 8.00E+00 | 1.01E+01 | 9.18E+00 |
|  |  |  |  |  |  |  |  |  |

^a^Log_10_ colony forming units g^-1^ x 10^n^.

Key of the media (target population): MCB, Medium for Colon Bacteria (total cultivable bacteria); MRSC; de Man, Rogosa and Sharpe with cysteine (lactobacilli); BIF, Bifidobacteria, (*Bifidobacterium* spp.); RCM, Reinforced Clostridium Medium (clostridia); EMB, Eosin Methylene Blue (Enterobacteriaceae); BP, Bacteroides and Prevotella medium (*Bacteroides* and *Prevotella* spp.); VA, Veillonella Agar (*Veillonella* spp.).

**Supplementary Table 3.-** Relative quantities of fecal microbial populations in the different equol status of the postmenopausal women treated with soy isoflavones of this study as determined by qPCR using universal and group-specific primers.

| Woman | Month | Microbial population^a^ | | | | | | |
| --- | --- | --- | --- | --- | --- | --- | --- | --- |
|  |  | Bifidobacteria | Lactobacilli | *Clostridium leptum* | *Clostridium coccoides* | *Bacteroides* | Enterobacteria | *Atopobium* |
|  |  |  |  |  |  |  |  |  |
| **WA** | 0 | 8.64E-02 | 9.52E-02 | 2.81E+01 | 4.11E+01 | 5.72E+00 | 0.00E+00 | 4.83E+00 |
|  | 1 | 2.26E-03 | 2.63E-03 | 2.99E+01 | 4.66E+01 | 5.13E+00 | 0.00E+00 | 6.53E+00 |
|  | 3 | 3.96E-03 | 4.24E-01 | 3.96E+01 | 2.89E+01 | 1.20E+01 | 0.00E+00 | 6.61E+00 |
|  | 6 | 2.12E-01 | 2.89E-02 | 3.45E+01 | 3.46E+01 | 9.08E+00 | 0.00E+00 | 4.30E+00 |
|  |  |  |  |  |  |  |  |  |
| **WB** | 0 | 2.15E+01 | 2.85E+00 | 1.08E+01 | 7.07E+00 | 7.80E+00 | 1.48E+00 | 4.40E+00 |
|  | 1 | 2.67E+01 | 3.81E-02 | 9.56E-01 | 1.32E+01 | 1.89E+01 | 8.11E-01 | 1.05E+01 |
|  | 3 | 3.29E+01 | 8.97E-02 | 4.26E+00 | 5.08E+00 | 8.15E+00 | 4.68E-01 | 5.58E+00 |
|  | 6 | 4.70E+01 | 5.37E-01 | 7.49E+00 | 7.94E+00 | 2.05E+01 | 3.77E-01 | 9.49E+00 |
|  |  |  |  |  |  |  |  |  |
| **WC** | 0 | 1.20E+01 | 1.87E+00 | 3.94E+01 | 7.37E+00 | 6.97E+00 | 1.64E-02 | 4.19E-01 |
|  | 1 | 1.87E+00 | 4.21E-04 | 1.64E+01 | 2.72E+00 | 1.80E+01 | 6.26E-03 | 3.08E+00 |
|  | 3 | 2.63E+00 | 6.73E-02 | 4.66E+01 | 7.38E+01 | 2.24E+01 | 1.54E-03 | 7.01E+00 |
|  | 6 | 1.59E+00 | 2.61E-02 | 4.77E+01 | 6.56E+01 | 8.17E+00 | 6.01E-05 | 3.98E+00 |
|  |  |  |  |  |  |  |  |  |
| **WD** | 0 | 1.08E+01 | 1.31E-02 | 2.88E+01 | 5.71E+01 | 6.36E+00 | 0.00E+00 | 4.13E+00 |
|  | 1 | 1.91E+00 | 3.12E+00 | 3.40E+01 | 4.89E+01 | 9.61E+00 | 0.00E+00 | 2.67E+00 |
|  | 3 | 6.97E+00 | 2.61E-03 | 2.60E+01 | 4.38E+01 | 8.59E+00 | 0.00E+00 | 3.11E+00 |
|  | 6 | 7.00E+00 | 2.18E+00 | 1.60E+01 | 5.13E+01 | 4.61E+00 | 0.00E+00 | 2.36E+00 |
|  |  |  |  |  |  |  |  |  |
| **WE** | 0 | 1.86E+00 | 2.18E-01 | 2.15E+01 | 5.54E+00 | 9.31E+00 | 2.24E-03 | 2.27E+00 |
|  | 1 | 3.16E+00 | 3.77E-02 | 3.45E+01 | 1.12E+01 | 1.08E+01 | 4.04E-04 | 1.00E+00 |
|  | 3 | 3.66E+00 | 2.37E-01 | 2.56E+01 | 6.16E+00 | 7.47E+00 | 4.11E-04 | 1.86E+00 |
|  | 6 | 3.51E+00 | 6.18E-02 | 3.25E+01 | 1.39E+01 | 7.75E+00 | 2.19E-04 | 1.13E+00 |
|  |  |  |  |  |  |  |  |  |
| **WF** | 0 | 2.30E-01 | 4.30E-03 | 2.46E+01 | 4.64E+01 | 2.71E+01 | 5.08E-03 | 8.54E-01 |
|  | 1 | 1.07E-03 | 4.04E-02 | 1.28E+01 | 7.22E+01 | 1.76E+01 | 1.33E-02 | 7.66E-01 |
|  | 3 | 2.18E-01 | 1.35E-02 | 2.99E+01 | 5.44E+01 | 2.41E+01 | 8.38E-04 | 1.33E+00 |
|  | 6 | 3.14E-01 | 3.46E-02 | 6.02E+00 | 6.33E+01 | 1.68E+01 | 1.29E-02 | 4.57E-01 |
|  |  |  |  |  |  |  |  |  |
| **WG** | 0 | 2.10E-01 | 4.42E-02 | 2.14E+01 | 2.71E+01 | 1.90E+01 | 2.98E-04 | 8.48E+00 |
|  | 1 | 7.82E-01 | 2.56E-03 | 2.18E+01 | 2.97E+01 | 1.93E+01 | 8.23E-05 | 8.07E+00 |
|  | 3 | 2.77E+00 | 1.31E-02 | 4.06E+01 | 1.79E+01 | 1.22E+01 | 1.83E-04 | 5.15E+00 |
|  | 6 | 1.21E+00 | 8.86E-02 | 3.48E+01 | 1.63E+01 | 7.53E+00 | 9.48E-03 | 7.21E+00 |
|  |  |  |  |  |  |  |  |  |
| **WH** | 0 | 1.23E+00 | 2.91E-02 | 3.17E+01 | 5.38E+00 | 2.18E+02 | 0.00E+00 | 5.49E-01 |
|  | 1 | 1.15E+01 | 1.99E-02 | 5.63E+01 | 2.31E+01 | 1.12E+02 | 0.00E+00 | 1.31E+00 |
|  | 3 | 9.46E+00 | 9.99E-02 | 5.01E+01 | 2.72E+01 | 4.97E+01 | 0.00E+00 | 2.78E+00 |
|  | 6 | 1.55E+01 | 1.20E+00 | 2.09E+01 | 1.57E+01 | 3.54E+01 | 0.00E+00 | 3.50E+00 |
|  |  |  |  |  |  |  |  |  |
| **WI** | 0 | 1.72E+00 | 9.21E-03 | 3.09E+01 | 9.52E+00 | 6.80E+00 | 0.00E+00 | 5.59E+00 |
|  | 1 | 7.25E+00 | 2.20E-02 | 1.89E+01 | 5.81E+00 | 5.34E+00 | 0.00E+00 | 1.03E+01 |
|  | 3 | 5.14E+00 | 5.66E-02 | 2.12E+01 | 6.14E+00 | 4.15E+00 | 0.00E+00 | 1.05E+01 |
|  | 6 | 4.33E+00 | 1.68E-02 | 2.52E+01 | 1.05E+01 | 5.59E+00 | 0.00E+00 | 3.95E+00 |
|  |  |  |  |  |  |  |  |  |
| **WJ** | 0 | 9.29E+00 | 3.25E-01 | 1.29E+01 | 3.41E+01 | 4.15E+00 | 2.33E-03 | 7.31E+00 |
|  | 1 | 5.97E+00 | 2.75E-01 | 1.41E+01 | 1.34E+01 | 2.97E+00 | 2.52E-03 | 1.37E+01 |
|  | 3 | 5.49E+00 | 6.79E-03 | 2.01E+01 | 6.67E+00 | 2.93E+00 | 2.24E-04 | 1.72E+01 |
|  | 6 | 9.42E+00 | 2.64E-02 | 2.12E+01 | 1.35E+01 | 3.62E+00 | 2.40E-03 | 5.95E+00 |
|  |  |  |  |  |  |  |  |  |
| **WK** | 0 | 1.30E+00 | 7.92E-02 | 3.45E+01 | 2.69E+01 | 0.00E+00 | 2.18E-02 | 4.41E+00 |
|  | 1 | 3.44E+00 | 3.58E-02 | 3.48E+01 | 2.43E+01 | 0.00E+00 | 7.09E-02 | 4.31E+00 |
|  | 3 | 9.08E-01 | 1.48E-02 | 3.19E+01 | 3.13E+01 | 0.00E+00 | 1.48E-02 | 7.36E+00 |
|  | 6 | 2.45E+00 | 6.36E-03 | 4.01E+01 | 2.52E+01 | 0.00E+00 | 2.27E-04 | 5.44E+00 |
|  |  |  |  |  |  |  |  |  |
| **WL** | 0 | 1.69E+00 | 1.39E-02 | 1.76E+01 | 2.67E+01 | 1.63E+01 | 7.22E-02 | 5.70E+00 |
|  | 1 | 1.14E-01 | 1.67E-02 | 1.93E+01 | 3.25E+01 | 2.70E+01 | 1.79E-01 | 6.25E+00 |
|  | 3 | 6.36E-02 | 8.13E-03 | 2.09E+01 | 4.02E+01 | 2.95E+01 | 2.28E-02 | 4.47E+00 |
|  | 6 | 1.93E+00 | 6.59E-02 | 2.10E+01 | 2.26E+01 | 1.33E+01 | 6.46E-04 | 8.12E+00 |
|  |  |  |  |  |  |  |  |  |
| **WM** | 0 | 1.32E+00 | 1.10E-01 | 3.75E+01 | 2.98E+01 | 5.13E+00 | 0.00E+00 | 3.87E+00 |
|  | 1 | 1.65E+01 | 2.02E-03 | 5.55E+01 | 2.81E+01 | 8.63E+00 | 0.00E+00 | 8.24E+00 |
|  | 3 | 2.19E+00 | 1.23E-03 | 2.93E+01 | 3.38E+01 | 1.96E+01 | 0.00E+00 | 1.29E+01 |
|  | 6 | 5.11E-01 | 1.41E-02 | 2.57E+01 | 2.70E+01 | 1.15E+01 | 0.00E+00 | 6.09E+00 |
|  |  |  |  |  |  |  |  |  |
| **WN** | 0 | 5.46E+00 | 2.61E-02 | 1.93E+01 | 3.30E+01 | 6.67E+00 | 2.60E-02 | 2.83E+00 |
|  | 1 | 1.16E+01 | 1.20E-02 | 1.61E+01 | 3.40E+01 | 3.50E+00 | 1.90E-02 | 4.71E+00 |
|  | 3 | 1.39E+01 | 4.62E-02 | 2.17E+01 | 2.64E+01 | 2.62E+00 | 6.17E+00 | 4.84E+00 |
|  | 6 | 2.17E+00 | 8.43E-03 | 1.38E+01 | 2.51E+01 | 4.13E+00 | 7.27E-04 | 3.41E+00 |
|  |  |  |  |  |  |  |  |  |
| **WO** | 0 | 3.26E+00 | 5.86E-02 | 1.67E+01 | 2.23E+01 | 1.22E+01 | 7.05E-04 | 3.37E+00 |
|  | 1 | 3.40E+00 | 5.09E-02 | 1.69E+01 | 1.68E+01 | 1.02E+01 | 7.48E-03 | 3.45E+00 |
|  | 3 | 1.24E+00 | 3.82E-02 | 1.56E+01 | 2.07E+01 | 1.41E+01 | 1.97E-03 | 2.97E+00 |
|  | 6 | 1.72E+01 | 2.84E-02 | 9.96E+00 | 1.50E+01 | 1.48E+01 | 5.59E-04 | 8.33E+00 |
|  |  |  |  |  |  |  |  |  |
| **WP** | 0 | 1.58E+00 | 7.95E-03 | 2.59E+01 | 1.79E+01 | 2.63E+02 | 0.00E+00 | 5.75E+00 |
|  | 1 | 1.33E+00 | 7.27E-03 | 1.05E+01 | 1.77E+01 | 3.77E+02 | 0.00E+00 | 4.88E+00 |
|  | 3 | 2.79E+00 | 2.41E-02 | 1.59E+01 | 4.08E+01 | 1.35E+02 | 0.00E+00 | 5.06E+00 |
|  | 6 | 2.73E+00 | 8.20E-03 | 1.56E+01 | 1.32E+01 | 4.29E+02 | 0.00E+00 | 3.32E+00 |
|  |  |  |  |  |  |  |  |  |

^a^% of the total bacterial 16S rDNA x 10^n^, as determined using the universal prokaryotic primers TBA-F and TBA-R (Table 1).

**Supplementary Figure 3.-** Principal Coordinate Analysis (PCoA) of the pooled microbial results obtained by culturing and qPCR and the equol production phenotype. Women 1, 9, 14 and 26, are equol producers and correspond to WA, WC, WG and WP in supplementary tables.
